# Impact of long COVID-19 on employees of the mining industry in South Africa: a cross-sectional study

**DOI:** 10.11604/pamj.2025.51.37.40739

**Published:** 2025-06-09

**Authors:** Henry Fomundam, Hanson Nyambi, Francis Hyera, Nkengafac Villyen Motaze

**Affiliations:** 1 Public Health and Pharmaceutical Care Innovations, Pretoria, Gauteng Province, South Africa,; 2 Department of Public Health, Faculty of Health Sciences, Walter Sisulu University, Mthatha, Eastern Cape, South Africa,; 3 Medicine Usage in South Africa, School of Pharmacy, Faculty of Health Sciences, North-West University, Potchefstroom, North-West Province, South Africa

**Keywords:** COVID-19, post-acute COVID-19 syndrome, SARS-CoV-2

## Abstract

**Introduction:**

coronavirus disease 2019 (COVID-19) was declared a pandemic in March 2020, and most patients recover within a few days or weeks. However, some patients suffer from long COVID, which is characterized by prolonged symptoms of varying severity. Given the importance of the mining sector to the South African economy, it is critical to comprehend the impact of long COVID on the mining sector.

**Methods:**

we carried out a cross-sectional study that included data extraction from participant medical records and responses to study questionnaires. We used unique identifiers to anonymize participant information and match data from medical records to participant questionnaires.

**Results:**

data from 239 medical records and 362 questionnaires were provided by employees from three mines. About 7% of people with COVID-19 were identified as having long COVID. In addition, there was no difference in productivity between people with and without long COVID.

**Conclusion:**

our findings are not generalizable to the entire mining sector due to the limited number of participants who provided data. Additional studies that include a larger sample size are required to obtain more robust estimates of the prevalence of long COVID in the mining industry and explore potential effects of various treatment interventions for patients with long COVID.

## Introduction

Coronavirus disease 2019 (COVID-19) is caused by the novel coronavirus called severe acute respiratory syndrome coronavirus 2 (SARS-CoV-2) [1]. Severe acute respiratory syndrome coronavirus 2 infection is usually mild with symptoms such as chills, fever, cough, sore throat, chills, loss of smell or taste, shortness of breath, headache, nausea, and diarrhea. Infected individuals can have no clinical disease, with up to 30% of cases being asymptomatic [2]. However, comorbidities including diabetes, obesity, and advanced age, amongst others, are risk factors for developing severe disease and death. People of all ages can be infected with SARS-CoV-2 [3], and cases with mild disease usually recover within 1-2 weeks [4]. The Centers for Disease Control and Prevention in the USA (US CDC) describe the presence of persistent symptoms or the occurrence of new or recurring symptoms four weeks or more after acute COVID-19 as a clinical syndrome called long COVID [5]. Other terms used to describe this condition include Post COVID condition, post-acute COVID-19, long-term effects of COVID, post-acute COVID syndrome, chronic COVID, long-haul COVID, late sequelae, post-acute COVID-19 syndrome, post-acute sequelae of SARS-CoV-2 infection (PASC), etc. Long haulers often present with fatigue, headache, dyspnoea, and anosmia [6]. The occurrence of Long COVID varies in different settings, from 57.00% (95% CI 56.59 to 57.43) in the UK [7] to 29.2% (95% CI: 25.3%,33.4%) in India [8]. Female sex, and multiple symptoms during acute COVID-19 [6] are risk factors for developing long COVID. Up to 76% of hospitalized patients have been reported to have symptoms six months after the onset of acute COVID-19, and individuals with mild disease can also develop long COVID [9]. Since the start of the pandemic in January 2020 and until September 2021, there was no universally agreed-upon definition of long COVID as a medical condition. In September 2021, WHO proposed a clinical case definition of post-COVID-19 condition for adults who have had probable or confirmed SARS-CoV-2 infection [10]. This definition identifies post-COVID-19 condition as occurring three months from the start of acute COVID-19, with symptoms that last two months or longer that cannot be explained by an alternative diagnosis [11].

Any individual who had acute COVID-19 is at risk of developing long COVID, but there are risk factors such as disease severity [12], sex, older age and high body mass index [6]. Prolonged treatment is required for long haulers with persistent clinical disease. This need for long-term treatment does not only increase the strain on health systems but also reduces the available workforce, potentially causing a decrease in productivity. The increasing prevalence of long COVID and the persistence of poor health in these long haulers led to the designation of long COVID as a disability in the USA [13] and inclusion of long COVID in several employee-related financial support schemes in the UK. It is likely that other countries will take similar steps given the substantial proportion of the workforce that will be affected by long COVID in the aftermath of the COVID-19 pandemic. The COVID-19 pandemic has had an impact on the global economy, with varying effects on different industries. An analysis of the impact of the COVID-19 pandemic in Europe found that the most substantial impact of COVID-19 on the mining sector was on feasibility studies, development of new mines and activities preceding closure of mines, with a smaller, short-term impact on production [14]. In Australia, scenario-based simulations predicted no significant impact of COVID-19 containment measures on the mining industry [15]. Consistent with these findings, a review of the global data in the mining industry found that COVID-19 will have a short-term impact on the mining sector [16]. Despite these observations, it is important to emphasize that COVID-19 affects the mining industry in different countries to various extents depending on national lockdown policies, occupational health conditions and commodities involved [17].

The South African mining industry was also affected by the pandemic. As of 3 May 2022, the Minerals Council of South Africa reported a total of 64,837 confirmed cases out of 450,000 employees in 385 mines [18]. Early in 2020, government shutdowns affected mining activities in South Africa and other mineral-producing countries [19]. By the end of the first quarter of 2021, South Africa had experienced a reduction in employment within the mining sector and a decrease in mining revenue as a result of lower outputs [20]. This loss of productivity was mainly due to a reduction in the workforce. There is limited data on the impact of COVID-19 on loss of productivity resulting from absenteeism and presentness in the mining industry. Several studies have demonstrated the beneficial impact of HIV treatment programs implemented in South African mines on employee absenteeism [21,22]. Consequently, it is important to understand the drivers of loss of productivity to implement tailored interventions. Long COVID is a recognized outcome of acute COVID-19 which requires long-term management of patients. The burden of long COVID and the extent to which long COVID impacts loss of productivity in the mining sector of South Africa are not known. This loss of productivity could be due to absenteeism, which is when employees are away from the workplace because of ill health or due to presentness, which is when employees are at the workplace but are not fully functional. The aim of this study was to estimate the prevalence of long COVID, assess the effects of long COVID on workforce productivity in the mining sector and identify interventions that improve the clinical course of long COVID.

## Methods

**Study design:** we conducted this study in South Africa, which is an upper-middle-income country. Three mining companies, each specialized in underground mining of either gold, platinum, or coal, were included, and for the purposes of confidentiality, we refer to them as “Mine A”, “Mine B” and “Mine C”.

**Study setting:** we conducted a cross-sectional descriptive study. Eligible participants completed a study questionnaire that was divided into two sections: Part A includes information on the acute COVID-19 illness and the duration of symptoms, which enabled identification of participants who had long COVID, while Part B was completed only by participants who reported persistence of symptoms following acute COVID-19 and therefore, were classified as having long COVID as defined by US CDC [5]. This section included questions on the impact of long COVID on work performance using the World Health Organization (WHO) health and work performance questionnaire (HPQ) [23] and questions on the effect of any treatment interventions received.

**Study participants:** we included employees of these companies who were aged 18 years and older, had a positive SARS-CoV-2 nucleic acid amplification test (NAAT) result obtained between May 2020 and July 2021, and provided written informed consent. In order to minimize bias, we selected a simple random sample of participants at each mine by generating random numbers and matching them to anonymized employee codes allocated by each mine. The questionnaire was administered by trained nurses who were employees of the respective mining companies. For study sites that had access to medical records of employees, a data extraction tool (MS Excel 2019 spreadsheet) was used to obtain additional data on participants. The sample size calculation was based on a 50% prevalence of long COVID (given that there were no estimates for long COVID in South Africa), an alpha value of 5%, and 80% power. This resulted in a sample size of 1902 participants from five mining companies who had initially agreed to participate in the study.

**Statistical analysis:** we used descriptive statistics for sociodemographic and clinical characteristics. Categorical variables were presented using absolute numbers and percentages with frequency tables used for data visualization. Treatment outcomes and interventions received by participants classified as having long COVID are detailed using tables. Missing data was excluded from the analysis. All data cleaning and analysis was done using R software for statistical analysis (v4.1.2; R Core Team 2021).

**Availability of data and materials:** the corresponding author will make the dataset used and/or analyzed for the study available upon reasonable request.

**Ethical clearance:** only de-identified data was collected from the study sites to ensure participant confidentiality. Furthermore, health personnel employed by the mining companies interviewed the participants and extracted data from the medical records. Prior to the start of data collection, ethical approval was obtained from the Human Research Committee of the Faculty of Health Sciences at Walter Sisulu University.

## Results

The three study sites submitted 239 participant medical records and 362 completed questionnaires (Table 1). There were 159 participants who responded to the questionnaires but had no data extracted from medical records and 36 participants with data extracted from medical records but for whom no questionnaire was submitted. The data extracted from the medical records was matched to the data from the questionnaires after the unique identifiers were harmonized. Data extracted from medical records is summarized in Table 2. The median age of participants was 42 years (range: 22-62), and the median BMI was 27 (range: 18 - 49). Symptoms of COVID-19 lasted five days on average (range: 1- 30), and admitted patients stayed in the hospital for about 11 days on average (range: 1 to 61). Most participants were male (86%), and Black Africans (94%) were the most prevalent race. The most common age group was individuals aged 36 to 45 years (45%), and 27% of those who reported on smoking were non-smokers, although smoking status was unavailable for 67% of participants. Symptomatic acute COVID-19 occurred in 58% of participants, and 22% of participants reported a pre-existing medical condition. In terms of hospital admission, 14% of the participants were hospitalized for COVID-19 treatment, while 84% of the participants were not admitted.

**Table 1:** number of participants submitted by study site

	Site Name	Number of employees	Confirmed COVID-19 cases*	Estimated sample size	Medical records	Interviews	Total
1	Mine A	40,536	5906	381	201	199	199
2	Mine B	20,903	5378	381	6	158	159
4	Mine C	22,000	1316	378	32	5	32
**Total**		**83, 439**	**12,600**	**1140**	**239**	**362**	**390**

* The total number of employees at each mine with laboratory-confirmed COVID-19 between May 01, 2020 and July 31, 2021; these numbers were provided by contact persons at the sites

**Table 2:** participant data extracted from medical records

Variable	Mine A, n=201	Mine B, n=6	Mine C, n=32	Total, N=239
**Sex at birth**				
Female	26 (13%)	4 (67%)	3 (9%)	33 (14%)
Male	175 (87%)	2 (33%)	29 (91%)	206 (86%)
**Age group**				
18 to 25	7 (3%)	1 (17%)	0 (0%)	8 (4%)
26 to 35	36 (18%)	1 (17%)	0 (0%)	37 (15%)
36 to 45	90 (45%)	1 (17%)	16 (50%)	107 (45%)
> 45	68 (34%)	3 (50%)	16 (50%)	87 (36%)
**Ethnic group**				
Black African	191 (95%)	3 (50%)	30 (94%)	224 (94%)
Coloured	0 (0%)	1 (17%)	0 (0%)	1 (0.3%)
White	10 (5%)	0 (0%)	2 (6%)	12 (5.0%)
Unknown	0 (0%)	2 (33%)	0 (0%)	2 (0.7%)
**Pre-existing medical condition**				
No	131 (65%)	0 (0%)	24 (75%)	155 (65%)
Yes	70 (35%)	4 (67%)	8 (25%)	82 (34%)
Unknown	0 (0%)	2 (33%)	0 (0%)	2 (1%)
**Smoking status**				
Non-smoker	36 (18%)	0 (0%)	28 (88%)	64 (27%)
Smoker	11 (5%)	0 (0%)	4 (12%)	15 (6%)
Unknown	154 (77%)	6 (100%)	0 (0%)	160 (67%)
**Admission status**				
Not admitted	168 (84%)	1 (17%)	32 (100%)	201 (84%)
Admitted	33 (16%)	1 (17%)	0 (0%)	34 (14%)
Unknown	0 (0%)	4 (67%)	0 (0%)	4 (2%)

**Prevalence of long COVID:** a total of 362 participants at the three sites provided responses to the questionnaires, and there were 25 cases of long COVID based on the duration of symptoms reported by the participants, corresponding to a 7% prevalence of long COVID. The prevalence of long COVID was highest at Mine C (20%), followed by Mine A (9.0%), and lowest at Mine B (3.8%).

**Duration and severity of long COVID:** out of the 355 participants, 109 had a severity of long COVID symptoms that was assessed using the participants' responses to items in the questionnaire that addressed the ease with which they carried out activities of daily life four weeks or more following the acute COVID-19 diagnosis. Among the 13 (56%) participants with long COVID who reported on their activities of daily life, eight (62%) reported being able to carry out their daily activities in the same manner as before their COVID-19 episode while five (38%) reported being able to better perform these activities after suffering from COVID-19. None of the participants reported that they were not able to perform activities of daily life as compared to the period before their COVID-19 episode, while 12 (48%) participants did not provide a response.

**Impact of long COVID on productivity:** self-reported impacts of long COVID are presented in Table 3. Absenteeism was assessed using the number of partial or full days of work that were missed by the participant due to issues related to their health. Among participants with Long COVID, all 22 participants who reported on the number of hours worked reported having worked for 40 hours during the previous week as expected by their employer. Presentness was measured using the participant´s self-reported work performance following the acute COVID-19 episode and self-rated performance during the month preceding the questionnaire completion. About 19% of employees reported that their performance was below average during the month preceding data collection compared to 27% of participants who reported below average performance during the period preceding the acute COVID-19 episode.

**Table 3:** self-reported impact of long COVID 4 weeks after acute COVID-19 diagnosis

Effects of long COVID	Total (N= 25)	Mine A (n= 18)	Mine B (n= 6)	Mine C (n= 1)
**Activities of daily life after acute COVID-19**				
Better	5 (38%)	3 (27%)	1 (100%)	1 (100%)
Same	8 (62%)	8 (73%)	0 (0%)	0 (0%)
Unknown	12	7	5	0
**Ability to work over previous week**				
better	6 (24%)	3 (17%)	2 (33%)	1 (100%)
same	17 (68%)	15 (83%)	2 (33%)	0 (0%)
worse	2 (8.0%)	0 (0%)	2 (33%)	0 (0%)
**Full workdays missed due to ill health**				
0	8 (53%)	4 (36%)	4 (100%)	0 (0%)
1	2 (13%)	2 (18%)	0 (0%)	0 (0%)
2	2 (13%)	2 (18%)	0 (0%)	0 (0%)
3	2 (13%)	2 (18%)	0 (0%)	0 (0%)
4	1 (8%)	1 (10%)	0 (0%)	0 (0%)
Unknown	10	7	2	1
**Half workdays missed due to ill health**				
0	7 (64%)	4 (57%)	3 (75%)	0 (0%)
2	1 (9%)	0 (0%)	1 (25%)	0 (0%)
3	1 (9%)	1 (14%)	0 (0%)	0 (0%)
4	1 (9%)	1 (14%)	0 (0%)	0 (0%)
6	1 (9%)	1 (14%)	0 (0%)	0 (0%)
Unknown	14	11	2	1
**Self-rated work performance prior to COVID-19**				
Above average	2 (9%)	1 (6%)	1 (20%)	0 (0%)
Average	14 (64%)	14 (82%)	0 (0%)	0 (0%)
Below average	6 (27%)	2 (12%)	4 (80%)	0 (0%)
Unknown	3	1	1	1
**Self-rated work performance in previous month**				
Above average	4 (19%)	3 (19%)	1 (20%)	0 (0%)
Average	13 (62%)	12 (75%)	1 (20%)	0 (0%)
Below average	4 (19%)	1 (6%)	3 (60%)	0 (0%)
Unknown	4	2	1	1

**Interventions for long COVID:** medical treatment, non-medical treatment and resting were used as interventions for long COVID (Table 4). A higher proportion of participants (87%) reported improvement following medical treatment, while two (13%) of those who received non-medical treatment reported improvement. Among 19 participants who rested as part of their treatment for long COVID, seven (37%) reported improved health, while 12 (63%) reported no improvement.

**Table 4:** treatment interventions received for long COVID

Treatment intervention	Total (N=25)	Mine A (n = 18)	Mine B (n = 6)	Mine C (n= 1)
**Medical treatment taken**				
No	3 (12%)	0 (0%)	2 (33%)	1 (100%)
Yes	22 (88%)	18 (100%)	4 (67%)	0 (0%)
**Non-medical treatment taken**				
No	18 (72%)	13 (72%)	4 (67%)	1 (100%)
Yes	7 (28%)	5 (28%)	2 (33%)	0 (0%)
**Improvement with medical or non-medical treatment**				
Medical	13 (87%)	10 (100%)	3 (60%)	0 (0%)
Non-medical	2 (13%)	0 (0%)	2 (40%)	0 (0%)
Unknown	10	8	1	1
**Rest as an intervention**				
No	6 (24%)	4 (22%)	1 (17%)	1 (100%)
Yes	19 (76%)	14 (78%)	5 (83%)	0 (0%)
**Improvement following rest**				
No	12 (63%)	8 (57%)	4 (80%)	0 (0%)
Yes	7 (37%)	6 (43%)	1 (20%)	0 (0%)
Unknown	6	4	1	1
**Self-medication taken**				
No	16 (64%)	11 (61%)	4 (67%)	1 (100%)
Yes	9 (36%)	7 (39%)	2 (33%)	0 (0%)
**Improvement following self-medication**				
No	3 (33%)	3 (43%)	0 (0%)	0 (0%)
Yes	6 (67%)	4 (57%)	2 (100%)	0 (0%)
Unknown	16	11	4	1

## Discussion

We obtained an estimated 7% prevalence of long COVID among employees of the mining sector in this study. This prevalence is lower than previously published estimates [7,8], although these studies were not conducted among employees of the mining sector. Despite the small sample size of this study, we were unable to find other estimates of the prevalence of long COVID in the mining sector. None of the participants identified as having long COVID worked fewer hours than was required by their employer during the period of the study. This is a surprising finding that could be explained by the small sample size. Shortly after the onset of the COVID-19 outbreak in South Africa, a survey of employees showed that most permanent employees who had reduced working hours had little change to their remuneration [23]. Reduced working hours by employees represent a loss of productivity, and it can be challenging to distinguish a reduction in working hours due to employee illness from the reduction in working hours caused by precautions taken due to the pandemic. Acute COVID-19 is widely recognized as a cause of absenteeism [24,25], and long COVID exacerbates loss of productivity due to the prolonged inability of employees to perform their tasks. A study conducted in the UK [26] reported that up to 45% of employees with long COVID worked reduced schedules as a result of their illness. Regarding presentness, most participants (62%) reported similar performance compared to the expected average over the previous month. Four (19%) participants reported lower performance following COVID-19-related illness, and four (19%) reported higher performance during the previous month. Self-rated performance is not the ideal method when assessing employee performance, given the subjectiveness of reporting due to perceived concerns of repercussions from employers. However, comparing usual performance with recent self-rated performance is an informative way to gain insights into the impact of a recent health event, such as long COVID, from the participants´ perspective.

However, comparing usual performance with recent self-rated performance is an informative way to gain insights into the impact of a recent health event, such as long COVID, from the participants´ perspective. There is currently no published study reporting on presentness among individuals with long COVID in the mining industry. There are several drivers of presentness depending on the specific context or type of work performed. The push towards working from home does not apply to all job categories, even in the mining industry. Individual needs, organization, and occupational factors influence employees´ decisions to work [27,28] even when feeling unwell, and this may exacerbate the effects of presentness. The responses regarding activities of daily life, absenteeism, and presentness indicate that most participants were not adversely affected by long COVID, despite the persistence of symptoms beyond four weeks of acute COVID-19 diagnosis. A possible explanation could be the small number of participants with long COVID in this study. Another possibility could be the tendency to report a state of well-being to the interviewer, who was employed by the same company as the participant. Perhaps the participants could be worried about the implications of reporting diminished physical abilities that could jeopardize their job security or loss of income. This can only be verified by a control group of participants who were interviewed by an independent interviewer, a comparison that was not possible in this study.

Management guidelines formulated by WHO recommend symptomatic treatment for patients with mild COVID-19, including the use of medicines for fever and pain. Three participants reported using medicines that fall into this category and two of them reported feeling relieved of their symptoms. This treatment was combined with herbal extracts in most cases. A total of 10 participants reported having used plants or plant extracts as self-medication for their long COVID symptoms and six indicated that they felt relieved following this treatment. This highlights the potential beneficial effects of home remedies and over-the-counter medicines in the treatment of long COVID. Symptomatic treatment is recommended by WHO for mild symptoms of COVID-19. A small clinical trial conducted in the USA found that herbal extracts such as essential oil blends [29] were associated with improved outcomes among patients with long COVID. It is important to further explore treatment options apart from conventional medical treatments for long COVID while ensuring robustness of the evidence to avoid exposing patients to harmful interventions. Following off-label use of medicines such as chloroquine, ivermectin and lopinavir/ritonavir for COVID-19, with no evidence of efficacy, WHO issued recommendations against these therapies [30].

**Strengths and limitations:** this study has several strengths. Firstly, we included participants from several mines that exploit different minerals. This implies that the results are not limited to a select group of individuals within the mining sector. Secondly, data was collected from the study participants and from medical records. This dual approach allowed for robust information on participants to be collected which limits the influence of interviewer and recall bias. The study participants reported their duration of symptoms, which led to the identification of individuals with long COVID. Given that symptoms of long COVID might be mild and not require admission to hospital, self-reported symptom duration could be a better way of identifying such patients as opposed to the use of medical records only. There are several limitations to this study. Firstly, only a few participants were included due to challenges with enrollment of participants and data extraction from medical records at study sites. This small sample size probably had an impact on the estimated prevalence of long COVID and made it impossible to infer associated risk factors. Secondly, the data collection process did not enable inclusion of a control group since the second part of the questionnaire, which collected data on the impact of long COVID on productivity and the impact of treatment interventions, was completed only by participants with long COVID. Without collecting information on productivity from participants who do not have long COVID, it was not possible to obtain a more accurate measurement of the impact of long COVID.

## Conclusion

The overall prevalence of long COVID among individuals who had COVID-19 in this study is 7% and individuals with long COVID did not report lower productivity following their COVID-19 illness. These findings are not generalisable to the entire mining sector due to the limited number of participants who provided data. However, our results provide insights into the prevalence and impact of long COVID in the mining sector of South Africa.

### What is known about this topic

- Long COVID is a well-described medical condition that occurs in individuals who were infected with the SARS-CoV-2 virus;
- The prevalence of long COVID varies in different population sub-groups.

### What this study adds

- This study provides the first estimation of the prevalence of long COVID in South Africa;
- This study found no impact of long COVID on self-reported productivity.

## References

[1] Hu B, Guo H, Zhou P, Shi ZL (2021). Characteristics of SARS-CoV-2 and COVID-19. Nat Rev Microbiol.

[2] Centers for Disease Control and Prevention (CDC) COVID-19 Pandemic Planning Scenarios.

[3] Huang DT, McCreary EK, Bariola JR, Wadas RJ, Kip KE, Marroquin OC (2021). The UPMC OPTIMISE-C19 (OPtimizing Treatment and Impact of Monoclonal antIbodieS through Evaluation for COVID-19) trial: a structured summary of a study protocol for an open-label, pragmatic, comparative effectiveness platform trial with response-adaptive randomization. Trials.

[4] Hua W, Xiaofeng L, Zhenqiang B, Jun R, Ban W, Liming L (2020). The Epidemiological Characteristics of an Outbreak of 2019 Novel Coronavirus Diseases (COVID-19-China, 2020. China CDC Wkly.

[5] Centers for Disease Control and Prevention (CDC) (2021). Post-COVID Conditions: Information for Healthcare Providers. CDC.

[6] Sudre CH, Murray B, Varsavsky T, Graham MS, Penfold RS, Bowyer RC (2021). Attributes and predictors of long COVID. Nat Med.

[7] Taquet M, Dercon Q, Luciano S, Geddes JR, Husain M, Harrison PJ (2021). Incidence, co-occurrence, and evolution of long-COVID features: A 6-month retrospective cohort study of 273,618 survivors of COVID-19. PLoS Med.

[8] Arjun MC, Singh AK, Pal D, Das K, Alekhya G, Venkateshan M (2022). Characteristics and predictors of Long COVID among diagnosed cases of COVID-19. PLoS One.

[9] Stavem K, Ghanima W, Olsen MK, Gilboe HM, Einvik G (2021). Persistent symptoms 1.5-6 months after COVID-19 in non-hospitalised subjects: a population-based cohort study. Thorax.

[10] Soriano JB, Murthy S, Marshall JC, Relan P, Diaz J V, Clinical WHO (2022). Review A clinical case definition of post-COVID-19 condition by a Delphi consensus. Lancet Infect Dis.

[11] Nalbandian A, Sehgal K, Gupta A, Madhavan MV, McGroder C, Stevens JS (2021). Post-acute COVID-19 syndrome. Nat Med.

[12] Civil Rights Division (2021). Guidance on Long COVID as a Disability under the ADA Section 504 and Section 1557.

[13] Gałas A, Kot-Niewiadomska A, Czerw H, Simić V, Tost M, Wårell L (2021). Impact of Covid-19 on the mining sector and raw materials security in selected European countries. Resources.

[14] Dixon JM, Adams PD, Sheard N (2021). The impacts of COVID-19 containment on the Australian economy and its agricultural and mining industries. Aust J Agric Resour Econ.

[15] Laing T (2020). The economic impact of the Coronavirus 2019 ( COVID-2019 ): Implications for the mining industry. Extr Ind Soc.

[16] Ramadoo I The impact of COVID-19 on employment in mining.

[17] Minerals Council South Africa COVID-19 Dashboard.

[18] Swart A Understanding the sector impact of COVID-19: mining and minerals.

[19] Ahadjie J, Gajigo O, Gomwalk D, Kabanda F (2021). Impact of COVID-19 on mining: case study of four African countries. African Development Bank.

[20] Jockers D, Langlotz S, French D, Bärnighausen T (2021). HIV treatment and worker absenteeism: Quasi-experimental evidence from a large-scale health program in South Africa. J Health Econ.

[21] Meyer-Rath G, Pienaar J, Brink B, Van Zyl A, Muirhead D, Grant A (2015). The Impact of Company-Level ART Provision to a Mining Workforce in South Africa: A Cost-Benefit Analysis. PLoS Med.

[22] Kessler RC, Barber C, Beck A, Berglund P, Cleary PD, McKenas D (2003). The World Health Organization Health and Work Performance Questionnaire (HPQ). J Occup Environ Med.

[23] Statistics South Africa (2021). Results from Wave 2 survey on the impact of the COVID-19 pandemic on employment and income in South Africa. Statistics South Africa.

[24] Groenewold M, Burrer S, Ahmed F, Uzicanin A, Free H, Luckhaupt S (2020). Increases in Health-Related Work place Absenteeism Among Workers in Essential Critical Infrastructure Occupations During the COVID-19 Pandemic-United States, April 2020. MMWR Morb Mortal Wkly Rep.

[25] Faramarzi A, Javan-Noughabi J, Tabatabaee SS, Najafpoor AA, Rezapour A (2021). The lost productivity cost of absenteeism due to COVID-19 in health care workers in Iran: a case study in the hospitals of Mashhad University of Medical Sciences. BMC Health Serv Res.

[26] Chartered Institute of Personnel and Development (UK) (2022). Working with long COVID. CIPD.

[27] Kinman Gail Grant C (2021). Presenteeism during the COVID-19 pandemic: Risk factors and solutions for employers.

[28] Ferreira AI, Mach M, Martinez LF, Miraglia M (2022). Sickness Presenteeism in the Aftermath of COVID-19: Is Presenteeism Remote-Work Behavior the New (Ab)normal?. Front Psychol.

[29] Hawkins J, Hires C, Keenan L, Dunne E (2022). Aromatherapy blend of thyme, orange, clove bud, and frankincense boosts energy levels in post-COVID-19 female patients: A randomized, double-blinded, placebo controlled clinical trial. Complement Ther Med.

[30] World Health Organization (WHO) (2022). Therapeutics and COVID-19: living guideline-3 March 2022.

